# Sap Flow Responses to Warming and Fruit Load in Young Olive Trees

**DOI:** 10.3389/fpls.2019.01199

**Published:** 2019-10-02

**Authors:** Andrea Miserere, Peter S. Searles, Guadalupe Manchó, Pablo H. Maseda, Maria Cecilia Rousseaux

**Affiliations:** ^1^Centro Regional de Investigaciones Científicas y Transferencia Tecnológica de La Rioja (CRILAR-Provincia de La Rioja-UNLaR- SEGEMAR-UNCa-CONICET), Anillaco, Argentina; ^2^Departamento de Ciencias y Tecnologías Aplicada, Universidad Nacional de La Rioja, La Rioja, Argentina; ^3^Facultad de Agronomía, Universidad de Buenos Aires, Buenos Aires, Argentina; ^4^Departamento de Ciencias Exactas, Físicas y Naturales, Universidad Nacional de La Rioja, La Rioja, Argentina

**Keywords:** fruit load, global warming, heating, open-top chamber, sap flow, xylem anatomy

## Abstract

Global warming will likely lead to temperature increases in many regions of South America where temperatures are already considered to be high for olive production. Thus, experimental studies are needed to assess how water use in olive trees may be affected by global warming. The objectives of this study were to (i) evaluate the response of olive tree sap flow, stomatal conductance, and xylem anatomy to elevated temperature and (ii) determine whether fruit load may affect the temperature responses. A warming experiment using well-irrigated olive trees (cv. Arbequina) in open-top chambers (OTCs) with two temperature levels was performed from fruit set to the end of fruit growth in two seasons. Temperature levels were a near ambient control (T0) and a treatment 4°C above the control (T+). Trees were in the chambers for either one (2015–2016) or two seasons (2014–2015, 2015–2016) and were evaluated only in the second season when all trees were 3 years old. Whole-tree sap flow on leaf area basis, stomatal conductance, and aspects of xylem anatomy were measured. Sap flow was slightly higher in T+ than T0 trees heated for one season early in fruit development (summer) likely due to the elevated temperature and increase in vapor pressure deficit. Later in fruit development (fall), sap flow was substantially higher in the T+ trees heated for one season. Total vessel number per shoot was greater in the T+ than the T0 trees at this time due to more small-diameter vessels in the T+ trees, but this did not appear to explain the greater sap flow. The T+ trees that were heated for two seasons had less fruit load than the T0 trees due to little flowering. In contrast to trees heated for one season, sap flow was less in T+ than controls late in fruit development the second season, which was likely related to lower fruit load. An independent experiment using untreated trees confirmed that sap flow decreases when fruit load is below a threshold value. The results emphasize that multiple, interacting factors should be considered when predicting warming effects on water use in olive orchards.

## Introduction

Global warming has already led to temperature increases around 1°C, and further increases are expected at an increasing rate for the coming decades (IPCC, 2014). This temperature increase together with changes in rainfall patterns will likely have a negative impact on crop production in semiarid and arid environments (Fereres et al., 2011; De Ollas et al., 2019). Olive (*Olea europaea*) is a widely cultivated fruit tree species in the semiarid Mediterranean Basin and has expanded considerably over the last few decades to new warm, dry regions in the southern hemisphere including parts of Argentina and Australia (Torres et al., 2017). In such regions, current high temperatures are associated with little flowering in some cultivars (Ayerza and Sibbett, 2001; Aybar et al., 2015), as well as reductions in oil yield and quality (Mailer et al., 2010; Rondanini et al., 2014; García-Inza et al., 2014, García-Inza et al., 2016). Manipulative, warming experiments have also shown that increasing temperatures by 3°C to 4°C above current levels is likely to be detrimental for yields in southern Spain and northwest Argentina (Benlloch-González et al., 2018; Miserere et al., 2018; Benlloch-González et al., 2019).

In semiarid and arid regions, competition for water between agriculture and other sectors of the society is of critical importance and will likely only increase in the future as water scarcity intensifies (Fernández and Torrecillas, 2012). Regional climate modeling has indicated that the combination of increasing temperature and decreased rainfall in much of the Mediterranean Basin will lead to a greater need for irrigation (Tanasijevic et al., 2014). Further modeling suggests that increasing CO_2_ concentrations may lessen expected increases in crop water use under high temperature conditions by reducing stomatal conductance (Lorite et al., 2018). Due to the number of uncertainties involved, manipulative field experiments of water use under increased temperatures would provide much needed information.

Tree transpiration depends on the available soil water, leaf area, and the atmospheric demand. In a 2-year field experiment using open-top chambers (OTCs), sap flow of heated grapevines was higher than that of control plants the first season due to greater chamber vapor pressure deficit (VPD) and leaf area (Bonada et al., 2018). However, sap flow was less in the heated grapevines the second season due to depletion of soil water. In 30-year-old Scots pine trees, sap flow was higher under long-term warming conditions because of increases in stomatal conductance and needle area (Kellomäki and Wang, 2000). In olive, the sap flow response to prolonged warming has not yet been addressed to the best of our knowledge.

Whole-tree transpiration in olive has been observed to be sensitive to current air temperature conditions in the field. Transpiration under well-irrigated conditions was found to represent approximately 70% to 80% of crop evapotranspiration in 7-year-old cv. Manzanilla fina trees in northwest Argentina with sap flow increasing linearly over a wide range of daily mean temperatures (13°C–32°C) (Rousseaux et al., 2009). Below mean daily values of 13°C, sap flow was minimal. Similarly, transpiration values in a mature cv. Picual orchard showed a linear relationship with mean daily air temperature when normalized for intercepted solar radiation (Orgaz et al., 2007). Under rainfed conditions or deficit irrigation, low soil water content would likely alter such relationships due to reductions in stomatal conductance and water potentials (Fernández et al., 1997; Cuevas et al., 2013; Chebbi et al., 2018).

Water lost from trees including olive is regulated in large part by stomatal aperture (Jones, 1998; Hernández-Santana et al., 2016). In the short term, stomatal conductance (*g*_s_) increased with temperature in poplar and loblolly pine when measured at a similar VPD under controlled conditions in well-watered plants (Urban et al., 2017). However, increases in temperature under field conditions are often accompanied by greater VPD. When atmospheric demand increases, *g*_s_ has been consistently shown to decrease in olive trees (Moriana et al., 2002; Rousseaux et al., 2008). During a 2-week-long heat wave event in Italy, *g*_s_ dropped considerably in young trees when midday temperatures reached 40°C and recovered quickly following the heat wave (Haworth et al., 2018). Nevertheless, information is lacking as to how *g*_s_ in olive trees responds to more prolonged warming.

In the long term, changes in xylem anatomy in response to growth conditions can modify hydraulic conductivity and ultimately transpiration rates (Maseda and Fernández, 2006; Hacke et al., 2017). Elevated temperature increased the stem conduit area and hydraulic conductivity of saplings from several temperate and boreal species in a prolonged field experiment, particularly in species that were near the colder limit of their natural distribution (McCulloh et al., 2016). Furthermore, an increase in vein density and a decrease in vein diameter were observed in leaves of *Arabidopsis* ecotypes from different latitudes with increasing growth temperature (Adams et al., 2016). In this same study, leaf transpiration increased linearly with vein density when plants were evaluated under similar growing conditions. Olive is a species with small-diameter vessels compared to some other fruit trees species such as orange (*Citrus sinensis*) (Fernández et al., 2006) and has a low vulnerability to xylem embolisms and loss of hydraulic conductivity under moderate water stress (Tognetti et al., 2009; Torres-Ruiz et al., 2013). Yet, the effects of warming on the xylem anatomy of olive are not currently known.

Fruit load is important to consider in fruit tree studies because fruit are a significant carbon sink and affect plant water relations (Grossman and DeJong, 1994; Naor et al., 2008). In olive, whole-tree transpiration measured using lysimeters increased linearly with fruit load (Bustan et al., 2016). Stomatal conductance has also been shown to increase with fruit load in olive field studies in some cases (Martín-Vertedor et al., 2011; Naor et al., 2013), but not in others (Proietti et al., 2006; Bustan et al., 2016). In a warming experiment, *g*_s_ was higher in heated grapevines compared to controls on days with high *g*_s_ (Sadras et al., 2012b). However, low fruit load may have reduced the positive impact of elevated temperature on *g*_s_. Given that warming can affect flowering in olive, fruit load should be carefully considered in whole-tree warming experiments.

The objectives of the present study were to (i) evaluate the responses of olive tree sap flow, stomatal conductance, and xylem anatomy to prolonged elevated temperature and (ii) determine whether fruit load may have affected the temperature responses. A warming experiment was conducted in which olive trees were grown either in control OTCs with near ambient air temperature or in heated OTCs that were several degrees above the control temperature for 5 months. An independent experiment using plants with a wide range of fruit load allowed for a more rigorous interpretation of the warming experiment.

## Materials and Methods

### Plant Material

Cv. Arbequina olive trees were grown in an open, field nursery at the experimental field station of CRILAR-CONICET in Anillaco, La Rioja, in northwestern Argentina (28° 48′ S, 66° 56′ W, 1325 masl). The region is adjacent to the Andes mountains and is hot and dry with an annual evapotranspiration of about 1,600 mm and annual precipitation of 100 to 400 mm (Gómez-del-Campo et al., 2010; Searles et al., 2011). Own-rooted trees obtained from cuttings of a mother tree (San Gabriel Nursery S.A.; La Rioja) were transplanted when they were 14 months old in October 2013 to 30-L plastic pots filled with a 5:2 sandy soil:perlite mix and irrigated using 2 L h^−1^ drip emitters. The estimated water requirements were based on a previously derived function between mean daily temperature and sap flow (Rousseaux et al., 2009). Additional irrigation (30%) was provided in order to account for water losses from soil evaporation. Fertilization with macronutrients (15 N: 15 P: 15 K) was performed manually at a monthly interval, and micronutrients (B, 0.02% by weight; Cu, 0.01%; Fe, 3%; Mn, 1%; Zn, 1%, Mo 0.007%) + nitrogen (2.8%) + magnesium (0.5%) were applied weekly (Aminoquelant minors, Brometan, Spain).

### Warming Experiment—Treatments

The warming experiment was conducted from final fruit set (December 1) to the end of fruit and vegetative growth (early May) during two growing seasons (2014–2015 and 2015–2016). The trees were warmed during either one or both growing seasons in OTCs (**Table 1**). All measurements presented in this study were performed the second season (2015–2016) when all trees were 3 years old. Trees receiving two seasons of temperature treatment were transferred to the OTCs for the first season of warming on December 2014. After approximately 5 months of warming, these trees returned to the adjacent field nursery in May 2015. The same group of trees was heated again in 2015–2016 along with a second group of previously unheated trees of the same age. Control trees were also placed in the OTCs in a similar manner both seasons.

**Table 1:** Description of the warming experiment in the open-top chambers (OTCs) during two growing seasons (2014–2015, 2015–2016).

Seasons heated in OTCs (#)	Location during the season (December–May)			Sap flow measurements
	**2013–2014**	**2014–2015**	**2015–2016**	**Day of the year**
One season	Nursery	Nursery	OTC	25–35, 74–82
Two seasons	Nursery	OTC	OTC	14–24, 83–108

Trees were transplanted to 30-L plastic pots on October 2013 and heated either one or two seasons. All physiological measurements were performed in 2015–2016 when the trees were 3 years old.

The two temperature levels evaluated using the OTCs were a control slightly above ambient air temperature (T0) and a warming treatment with a target temperature set at 4°C above the control (T+). There were four OTCs per temperature level in a randomized complete block design with two factors (temperature and number of seasons heated in the OTCs). Blocks were used to account for any variability between OTCs in plant response that could have been related to the prevailing wind direction or minor differences in the heating system setup. Each OTC was designed to hold up to four trees, but only two trees per OTC were utilized in this study. One tree received its first season of warming, and the other received its second season. All trees were placed in cavities of about 30-cm soil depth with the soil surface in the pot being at the same level as the surrounding soil to avoid an increase in root temperature.

All OTC sidewalls (1.5 m each side and 2 m tall) were covered with 150-μm-thick, translucent polyethylene with low infrared transmittance (Premium Thermal Agrotileno PLDT221510; AgroRedes, Argentina). The T0 OTCs had some passive heating from the sidewalls but were not actively heated. The T+ OTCs had two complementary, active heating methods to increase temperature: a 6-m-long plastic sleeve with blackened stones through which heated air was sucked into the OTCs by fans during the daylight hours and an electric space heater (model AX-CA-1900 W; Axel, Argentina) whose operation was crucial during the night. Heated air from both methods entered into the OTCs *via* a PVC pipe with the pipe outlet positioned in the middle of the chamber at a height of 30 cm from the ground surface. Air flow from the outlet was redirected throughout the chamber by an air baffle. The electric heater was regulated by an electronic control system to avoid overheating (Cavadevices, Argentina). Shielded temperature sensors (TC1047A, Microchip Inc., China) were placed in each OTC at tree crown height (1.0 m), and the sensors were attached to a data logger recording every 15 min. The control program turned on or off the electric heater according to the 4°C differential target between T0 and T+ OTCs. Further details of the OTC design and function can be found in Miserere et al. (2019).

In addition to the air temperature readings from each OTC, relative humidity (RH) was recorded every 30 min using one sensor located at tree crown height in a T0 OTC and another in a T+ OTC. The sensors were moved every 2 to 3 days to a new pair of OTCs, and VPD was calculated based on the RH and temperature data (Allen et al., 1998). Outside of the chambers, air temperature and photosynthetic photon flux density (PPFD) were also monitored every hour in the field nursery adjacent to the OTCs. The PPFD was measured at a height of 3 m above the nursery trees (sensorPAR; Cavadevices), and air temperature was measured at tree crown height as was done within the OTCs. The PPFD inside the OTCs was measured periodically with a 1-m-long light bar (Cavadevices) and was about 75% of the PPFD above the field nursery due to absorption by the polyethylene walls and metal OTC structure with no differences between T0 and T+ OTCs.

### Warming Experiment—Plant Measurements

All fruit on each tree were harvested at the end of the season (April 29 2016) to determine fruit number and total fresh fruit weight per tree. On May 10, all leaves were removed from each tree to obtain leaf biomass after drying at 70°C until constant weight was reached in an oven, and specific leaf mass was calculated from leaf disks of known area sampled from 50 leaves per tree. Leaf area per tree was then estimated by dividing leaf dry weight per tree by specific leaf mass. Lastly, fruit load was calculated as fruit number per tree divided by leaf area.

Sap flow of the main trunk was determined using the heat balance method (Flow 32, Dynamax Inc., TX, USA). This method consists of applying a known amount of heat (Pin) to the entire trunk perimeter and measuring the vertical (up and down; Qu and Qd) and radial (Qr) dissipation of heat using several sets of thermocouples. The heat dissipated by sap flux (Qf) is then calculated by subtraction (Equation 1). The flux rate (F) is Qf divided by the average difference in temperature (dT) between upstream and downstream thermocouples and the heat capacity of water (Cp) (Equation 2).

(1) Qf=Pin−Qu−Qd−Qr

(2) F=Qf/(Cp*dT)

Given that the above method integrates the entire trunk, azimuth variations that often occur in sap flux measurements (López-Bernal et al., 2010) should be largely eliminated.

Measurements were conducted in both January (summer) and April (fall) (**Table 1**). In January, sap flow was measured for 10 consecutive days in the trees heated two seasons (2014–2015; 2015–2016) and their corresponding controls. One tree per OTC was measured including a total of four trees in T+ OTCs and four trees in T0 OTCs. Because only eight sensors were available due to economic limitations, the trees heated one season were measured for a similar 10-day period immediately following the two-season trees. In April, similar measurements were conducted, but the one-season trees were measured first. Measurements on the same tree were conducted over a limited number of consecutive days in order to better allow for comparisons between one- and two-season trees and to avoid possible damage to the trunk given that olive tree trunks can be damaged by heating (i.e., cracking) over extended periods (Rousseaux et al., 2009). Sensors were installed on trees with trunk diameters ranging from 20 to 30 mm (model SGB 16 and SGB25). The trunks were cleaned prior to installation, and canola oil was sprayed lightly on the trunk to improve contact between the trunk and the sensor. Power supply was adjusted daily by changing the heater input (0.25–0.3 W) to keep the average difference in temperature between upstream and downstream thermocouples (dT) between 0.7°C and 5°C. The dT was minimum at midday when sap flow was high and maximum at dawn when sap flow was negligible. Although the trunk was heated at night, this occurred in both the control and T+ trees. All sensors were heavily insulated and at least 15 cm from the soil surface to reduce heat flux from the soil. The sensors were connected to a Campbell CR10X data logger (Campbell Scientific, Logan, UT, USA) with readings taken every 60 s and averaged over 15 min. Daily sap flow was calculated as the accumulation of sap flow values along the day and expressed on a leaf area basis.

Stomatal conductance (*g*_s_) was measured at least once during each measurement period on leaves from both the trees heated one or two seasons using a portable porometer (model AP4; Delta-T Devices Ltd, UK). Two sunlit, fully expanded leaves were measured per tree on shoots with no fruit (i.e., vegetative shoots) for each measurement date. Additionally, leaves from shoots with fruit (reproductive shoots) were measured on some occasions. Measurements were performed at midday (13:00 to 14:00 h solar time) when temperatures were near their maximum daily values to coincide with the time of day when sap flow was likely highest, although it is recognized that maximum *g*_s_ most often occurs at midmorning (Moriana et al., 2002). The porometer was calibrated on each measurement date within both the T0 and T+ OTCs to properly reflect the temperature and humidity conditions. Potential oscillations in *g*_s_ were not directly considered (López-Bernal et al., 2018), but sap flow monitored at 5-min intervals on one day did not detect any significant oscillations.

Xylem anatomy including the diameter and number of vessels was evaluated in one current-year shoot from each of the one- and two-season trees heated in the 2015–2016 growing season. The shoots were collected at predawn in the OTCs at the end of the season (2015–2016). In the laboratory, the shoots were introduced in humidified labeled nylon bags and kept in the refrigerator until processing. Four cross sections from each shoot were submerged in a 10% sodium hypochlorite solution, washed with distilled water, and stained with safranin (1%) (Zarlavsky, 2014). Then, the cross sections were mounted on a slide with Canadian balsam. The cross sections were observed with an optical microscope (Carl Zeiss Axiostar Plus, Germany) connected to a digital camera (Canon Power Shot G9, Japan). ImageJ software was used for the image processing (version 1.50i; National Institutes of Health, USA).

### Fruit Load Experiment

To better interpret the warming experiment, eight olive trees from the field nursery with a wide range of fruit number per tree (33–900 fruit tree^−1^) were used to evaluate sap flow and *g*_s_ responses to fruit load (# m^−2^ leaf area). The different fruit numbers per tree were naturally obtained because of differences in flowering intensity and fruit set. The trees in the nursery were of the same age and characteristics as those used in the warming experiment. To determine fruit load, fruit and leaf number were counted visually on the field nursery trees in February 2016. These values were corroborated at the end of the season (April 20, 2016) by harvesting the fruit and removing the leaves from each of the eight trees.

Sap flow was measured on each tree between February 23 and March 13 in 2016. Only 13 days were included in the analyses because of electrical outages. Protocol for the measurements was the same as that of the warming experiment. On March 1, *g*_s_ was measured on three sunlit, fully expanded leaves of both vegetative and reproductive shoots per tree between 13:00 and 14:00 h solar time.

At the end of the season (April 20, 2016), a number of yield-related variables were measured in the eight trees including total fresh fruit weight, individual fruit dry weight, maturity index, and oil concentration (%). Individual fruit weight and maturity index were obtained using 50 fruit per tree when sufficient fruit were available. The fruit maturity index was calculated using the standard color evaluation of the skin and flesh with a 0- to 7-point scale (Uceda and Hermoso, 2001). Oil concentration (%) was ascertained from dried fruit (50) by nuclear magnetic resonance (model SLK-200; Spinlock, Argentina).

### Statistical Analyses

Most variables from the warming experiment such as fruit number, leaf area, *g*_s_, and number of vessels were analyzed using an ANOVA for fixed effects and a Duncan post-test to determine differences among treatment means (*p* ≤ 0.05). Assumptions of homogeneity of variances and normality of the data were previously confirmed using Levene and Shapiro-Wilk tests, respectively. The analysis of variance was performed in InfoStat statistical software (Di Rienzo et al., 2016). Linear and bilinear functions between mean daily temperature and response variables such as daily sap flow were determined in the warming experiment. Differences between the slopes of mean daily temperature and sap flow were evaluated using the Student *t* test. The significant functions presented in the tables and figures correspond to the highest *r*^2^ value for a particular variable. A similar approach was utilized in the fruit load experiment. Linear regression or bilinear regressions were analyzed with GraphPad Prism version 6.01 software (GraphPad Prism Software, Inc., LaJolla, CA, USA).

## Results

### Warming Experiment

**Figure 1:**
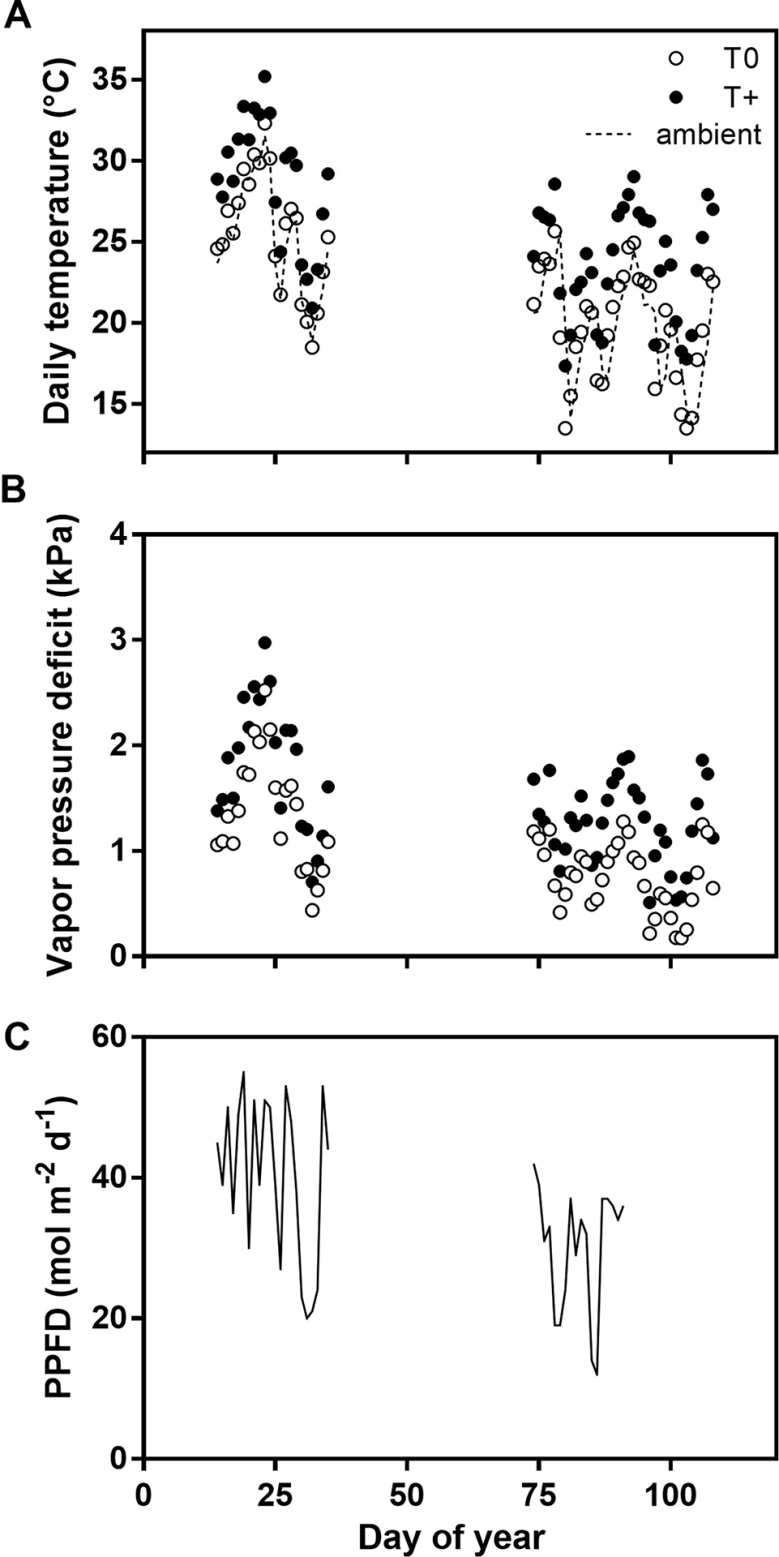
Mean daily temperature **(A)**, mean daily vapor pressure deficit **(**VPD; **B)**, and daily integrated photosynthetic photon flux density **(**PPFD; **C)** during the sap flow measurement periods of the warming experiment from January to April 2016. The daily temperature and VPD values were measured inside the control (T0) and heated (T+) open-top chambers (OTCs). Symbols represent the average of four independent OTCs. Ambient temperature values outside the OTCs are also shown with a dotted lined, and PPFD was measured only outside the OTCs. PPFD values were not available for the latter part of April.

Mean daily, ambient air temperature outside the OTCs was high during the January sap flow measurement period in the summer (18°C–32°C) and somewhat lower in April in the fall (13°C–26°C) (**Figure 1A**). The daily temperature in the experimental OTCs varied in accordance with the ambient air temperatures, and the daily mean temperature was 0.4°C and 3.9°C above the ambient temperature in the T0 and T+ OTCs, respectively. Mean daily VPD values were about 0.4 to 0.5 kPa greater in the T+ OTCs than in the T0 OTCs in both January and April (**Figure 1B**). Maximum VPD values were 6.3 in T+ and 5.0 kPa in T0 during the early afternoon of January 23. Daily PPFD values outside of the OTCs during the sap flow measurements varied between 20 and 53 mol m^−2^ d^−1^ in January and 16 to 42 mol m^−2^ d^−1^ in April for the dates available (**Figure 1C**). The lower values in April were due to the combined effect of lower solar elevation and shorter days.

**Figure 2:**
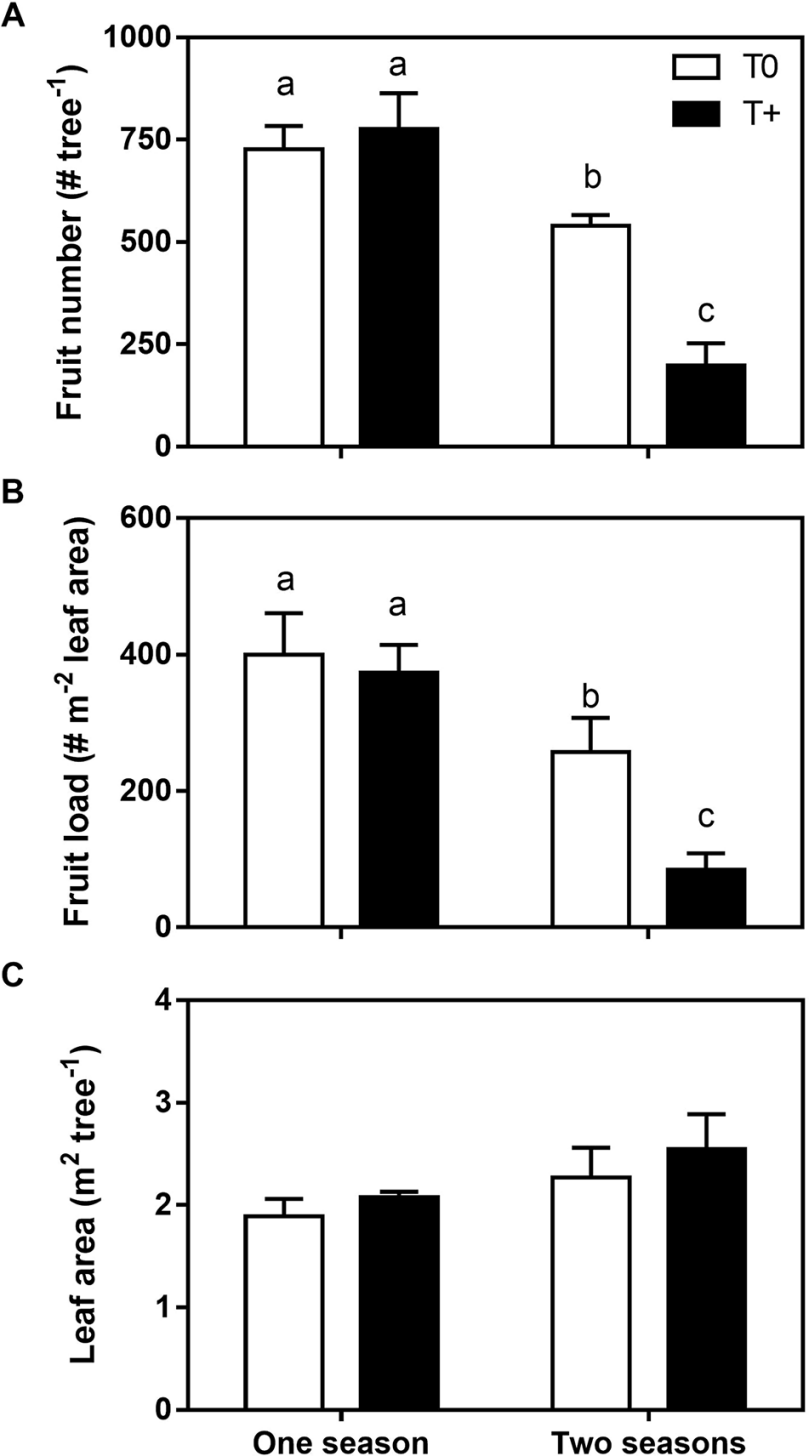
Fruit number per tree **(A)**, fruit load **(B)**, and leaf area per tree **(C)** at harvest in 2016 for trees in the control (T0) and heated (T+) open-top chambers (OTCs). The trees were treated in the OTCs either one (2015–2016) or two seasons (2014–2015, 2015–2016). All trees were3 years old during 2015–2016. Bars represent the averages ± one standard error (n = 4). Different letters indicate statistical significant differences between means (*p* ≤ 0.05).

At final harvest, fruit number averaged 750 fruit per tree in both T0 and T+ OTCs for trees that were treated only one season in 2015–2016 (**Figure 2A**). In contrast, trees growing in the OTCs for two seasons (2014–2015, 2015–2016) had significantly lower fruit numbers following the first season. Average fruit number was 540 fruit tree^−1^ in T0 and only 200 fruit tree^−1^ in T+. Given that fruit number was similar in the T0 and T+ trees in the first season since warming started after final fruit set in the summer, the reduced flowering intensity in the T+ trees the second season did not appear to be directly related to differences associated with alternate bearing. Fruit load (# m^−2^ leaf area) showed a similar pattern (**Figure 2B**) since leaf area at the end of the season was not affected by warming in either trees with one or two seasons of temperature treatment (**Figure 2C**).

**Figure 3:**
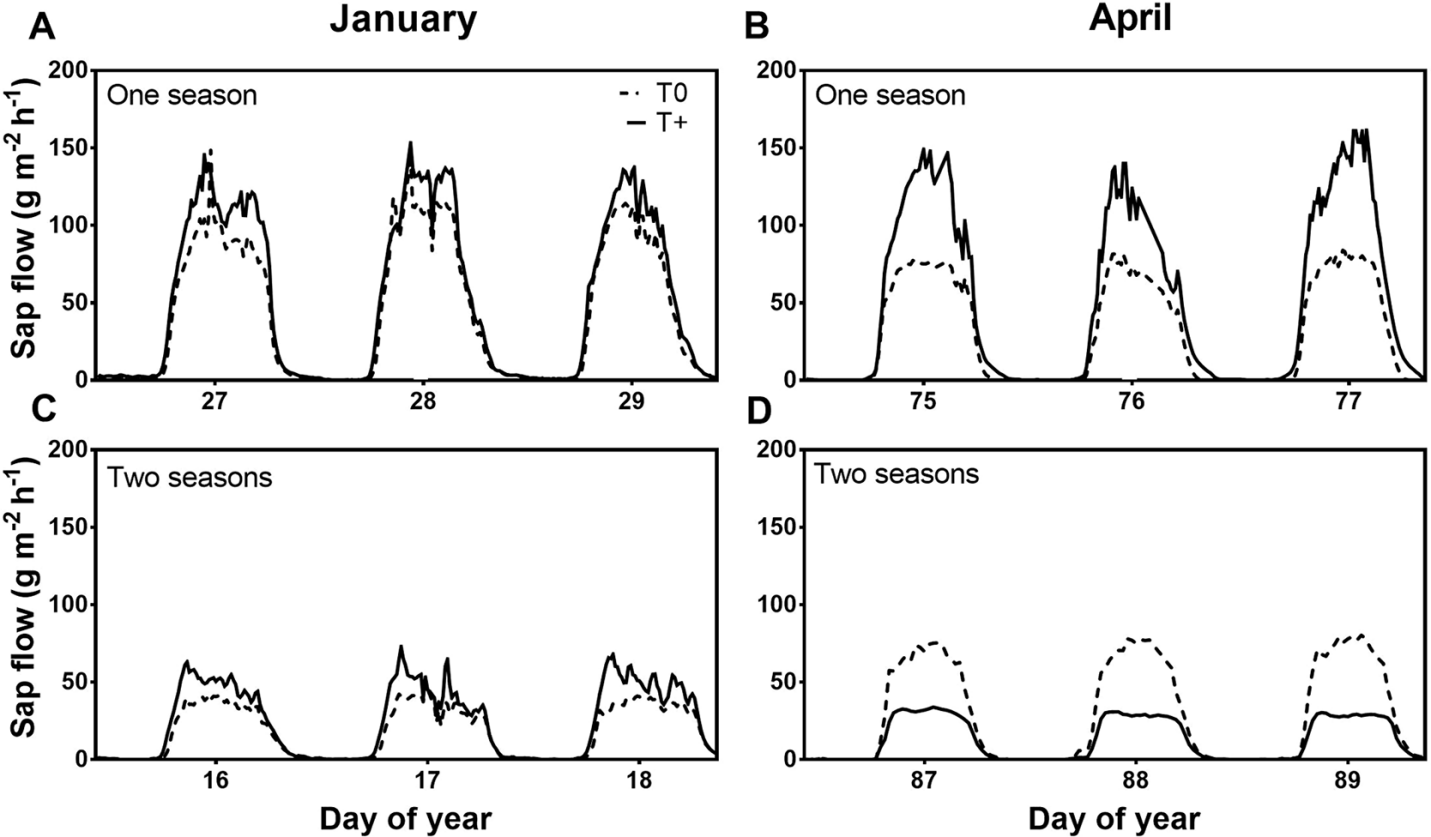
Diurnal sap flow per tree in January **(A**, **C)** and April **(B**, **D)** 2016 for 3 consecutive days in control (T0) and heated (T+) open-top chambers (OTCs). The trees were treated in the OTCs either one (2015–2016) or two seasons (2014–2015, 2015–2016). All trees were 3 years old during 2015–2016. Each line represents the average of four OTCs.

Diurnal sap flow patterns per unit of leaf area showed a sharp increase in the early morning with maximum rates between 10 and 16 h solar time, followed by a decrease until 20 h when sap flow became negligible (*p* ≤ 0.05; **Figure 3**). Regardless of being in either T0 or T+ OTCs, trees with one season in the OTCs and high fruit load (**Figure 3A**) had consistently higher sap flow values at midday than trees with two seasons in the OTCs and lower fruit load in January (**Figure 3C**; *p* ≤ 0.01). Although these measurements were conducted on different dates, mean ambient daily temperature was similar for the one- (25.7°C) and two-season (25.9°C) trees (**Figure 1**). With respect to warming, the T+ trees had slightly higher sap flow rates than T0 trees in January early in fruit development for both trees in the OTCs either one or two seasons (**Figures 3A, C**; *p* ≤ 0.10). Later in fruit development (April), the results were different. T+ had much higher sap flow than T0 in the one-season trees despite fruit load being the same in both OTC types (**Figure 3B**; *p* ≤ 0.01), while T+ had lower sap flow than T0 in the two-season trees (**Figure 3D**; *p* ≤ 0.01). In this latter case, fruit load was significantly lower in the T+ trees.

**Figure 4:**
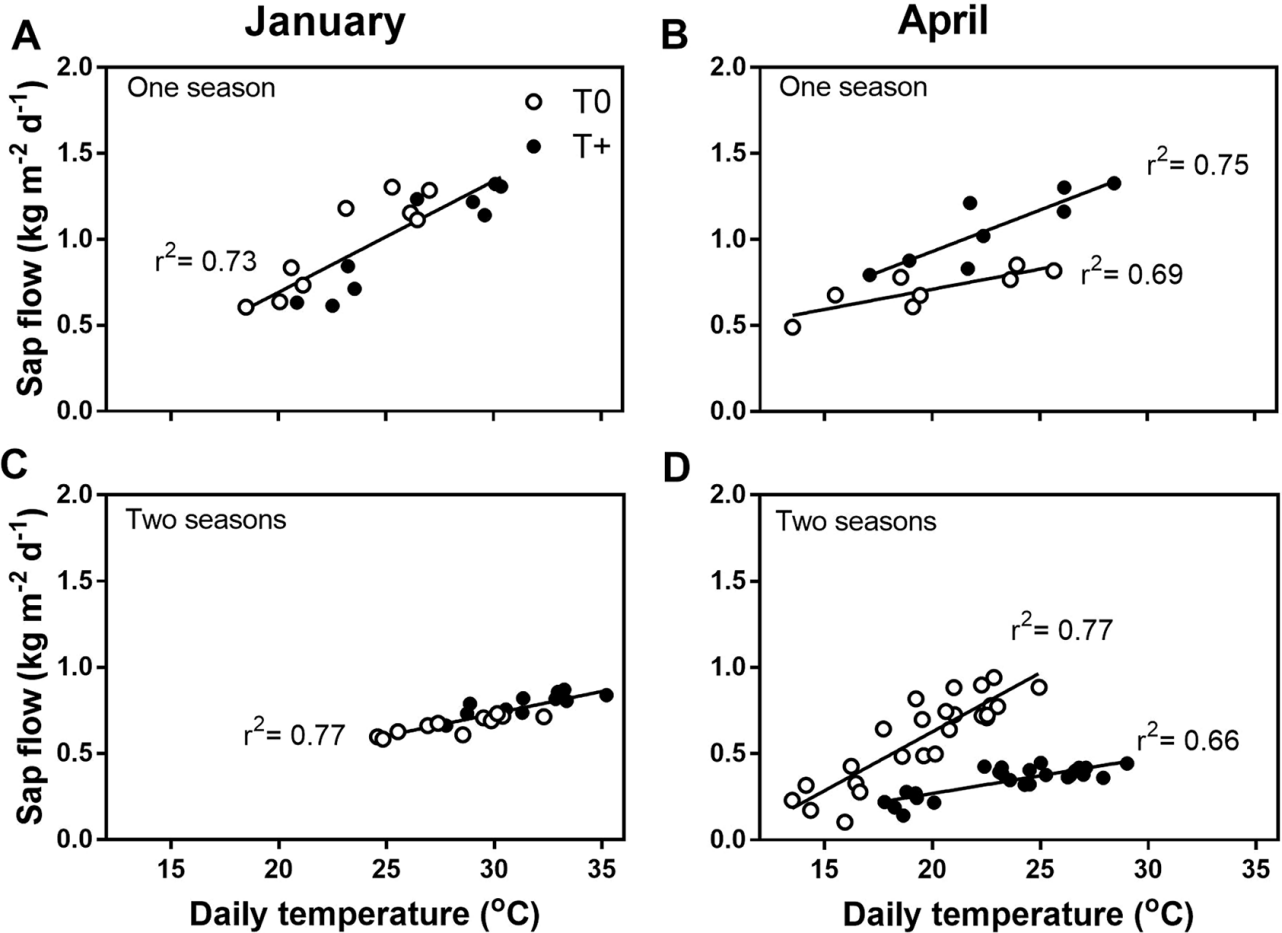
Daily sap flow per tree in January **(A**, **C)** and April **(B**, **D)** 2016 as a function of mean daily temperature in control (T0) and heated (T+) open-top chambers (OTCs). The trees were treated in the OTCs either one (2015–2016) or two seasons (2014–2015, 2015–2016). All trees were 3 years old during 2015–2016. Each point represents the average of four OTCs. Different regression lines are shown when the slopes are significantly different between the T0 and T+ data (*p* ≤ 0.05).

Daily sap flow per unit leaf area of T0 and T+ trees was explained by a single linear relationship with mean daily temperature in January in both one- and two-season trees (**Figures 4A, C**). In contrast, daily sap flow of T+ trees increased linearly at twice the rate of T0 plants (0.048 vs. 0.023 kg m^−2^ d^−1^ °C^−1^; *p* ≤ 0.05) in one-season trees in April (**Figure 4B**). Thus, T+ sap flow was higher than that of T0 for a given mean daily temperature. However, as suggested by the diurnal sap flow pattern in **Figure 3D**, the daily sap flow of T+ trees increased linearly with mean daily temperature in April, but at a rate three times less than T0 trees (0.021 vs. 0.068 kg m^−2^ d^−1 o^C^−1^; *p* ≤ 0.05) (**Figure 4D**). Lastly, mean daily temperature was highly correlated with mean daily VPD (*r* = 0.93), maximum daily temperature (*r* = 0.91), and maximum daily VPD (*r* = 0.90). For this reason, relationships between daily sap flow and these variables were similar to the relationships shown for mean temperature (data not shown).

Stomatal conductance of leaves from vegetative and reproductive shoots was similar between T0 and T+ for one-season trees, which all had high fruit load (**Table 2**). In contrast, *g*_s_ of leaves from vegetative and reproductive shoots was generally lower in T+ than T0 for two-season trees (*p* ≤ 0.05). As mentioned earlier, fruit load was low in the T+ trees and intermediate in the T0 trees. However, no differences were apparent in *g*_s_ values between the two shoot types for these same trees.

**Table 2:** Stomatal conductance (mmol m^−2^ s^−1^) at midday of leaves on vegetative and reproductive shoots in control (T0) or heated (T+) open-top chambers.

		Vegetative shoots		Reproductive shoots	
		One season	Two seasons	One season	Two seasons
January	T0	329 ± 22	405 ± 32 a	320 ± 11	371 ± 62
	T+	379 ± 26	269 ± 41 b	365 ± 59	191 ± 10
April	T0	377 ± 25	460 ± 50 a	318 ± 9.5	465 ± 12 a
	T+	325 ± 22	258 ± 78 b	290 ± 2.5	131 ± 30 b

The trees in the OTCs were heated one or two seasons. Means are shown ± standard error (n = 4). Different letters indicate statistically significant differences between means (p ≤ 0.05).

**Figure 5:**
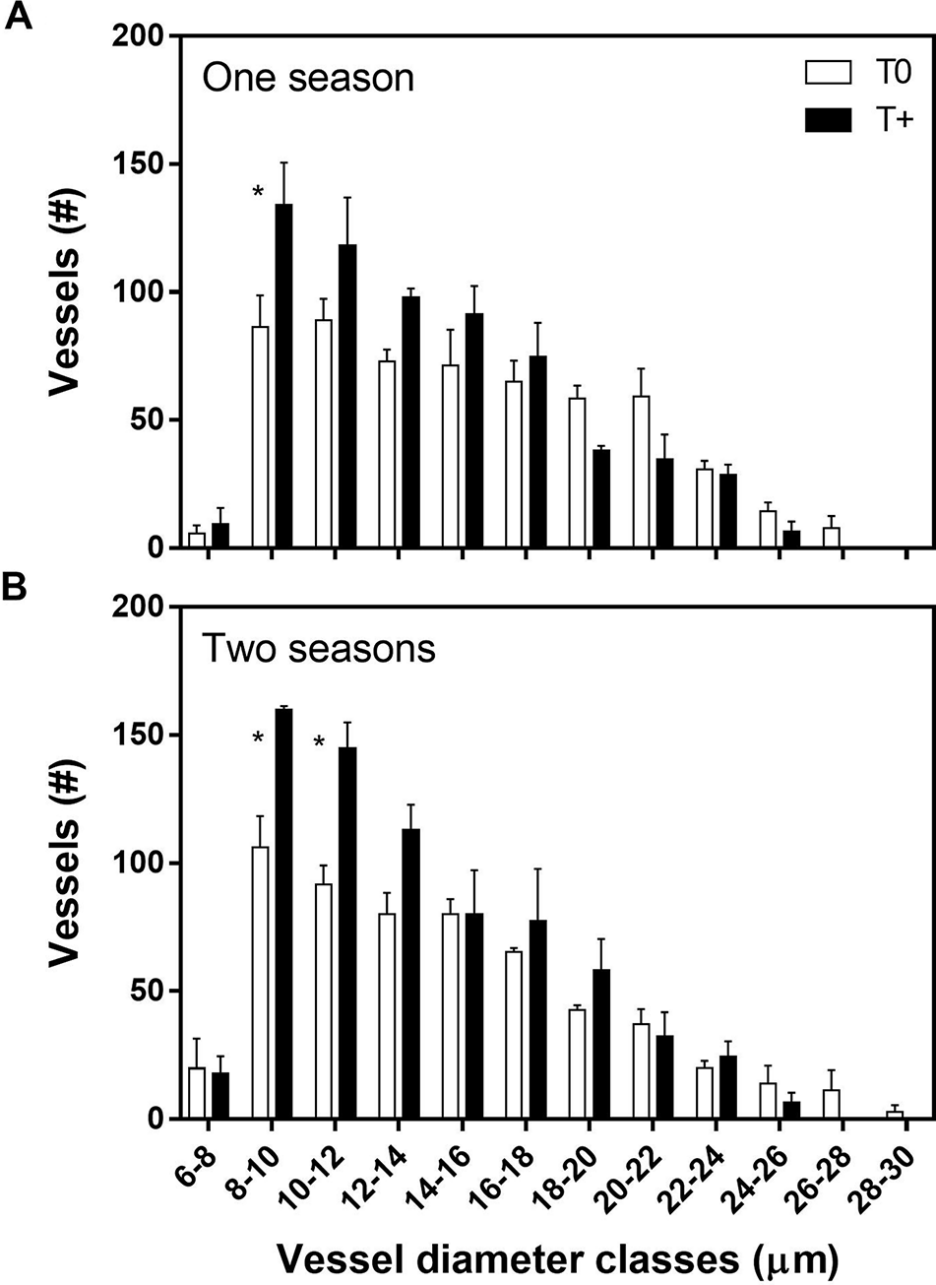
Vessel diameter distribution in 2016 for current-season shoots in control (T0) and heated (T+) open-top chambers (OTCs). The trees were treated in the OTCs either one (2015–2016; **A**) or two seasons (2014–2015, 2015–2016; **B**). All trees were 3 years old during 2015–2016. Bars represent the averages ± one standard error (n = 4). Asterisks between bars indicate statistically significant differences between means for a given vessel diameter class (*p* ≤ 0.05).

With respect to xylem anatomy, T+ trees had a greater number of small-diameter vessels than T0 in both one- and two-season trees (**Figure 5**; *p* ≤ 0.05), and only T0 had vessels in the largest size classes. Furthermore, the total number of vessels per shoot was significantly greater in the T+ trees (634 vessels for the entire cross-sectional area of the shoot) than the T0 trees (561) for one season (**Figure 5A**). Shoot cross-sectional areas averaged 3.15 and 3.65 mm^2^ in the T0 and T+ trees, respectively. A similar response occurred for the two-season trees (T+, 715; T0, 571) with cross-sectional areas of 2.86 in T+ and 3.51 mm^2^ in T0 (**Figure 5B**).

### Fruit Load Experiment

As was expected, the wide range of fruit load on these young trees strongly affected most yield-related variables (**Table 3**). Total fresh weight per tree increased with fruit load up to reaching a plateau at 200 fruit m^−2^ of leaf area above which no further increase occurred. Fruit maturity index decreased bilinearly with increasing fruit load, while individual fruit dry weight decreased linearly. Fruit oil concentration (%) was constant with fruit loads lower than 145 fruit m^−2^ and then decreased linearly as fruit load increased.

**Table 3:** Yield-related variables at harvest of the fruit load experiment in 2016. Fruit load was measured as the fruit number divided by the leaf area per tree (# m^−2^).

Fruit load (# m^−2^)	Total fruit fresh weight (g tree^−1^)	Maturity index (0–7)	Fruit dry weight (g fruit^−1^)	Fruit oil concentration (%)
8	58	6.5	1.37	43.8
9	96	6.0	1.25	42.0
49	450	5.1	1.21	43.9
144	1200	4.5	1.26	44.3
180	1348	4.2	1.19	42.7
250	1348	2.6	0.97	41.0
446	1808	1.5	0.78	36.8
613	1608	2.1	0.61	35.6
Function	Bilinear	Bilinear	Linear	Bilinear
*r*^2^	0.96	0.82	0.94	0.96

The data in each row represent an individual tree. The r^2^ values for the best modeled functions between fruit load and a given variable are shown when significant (p ≤ 0.05) at the bottom of the table.

**Figure 6:**
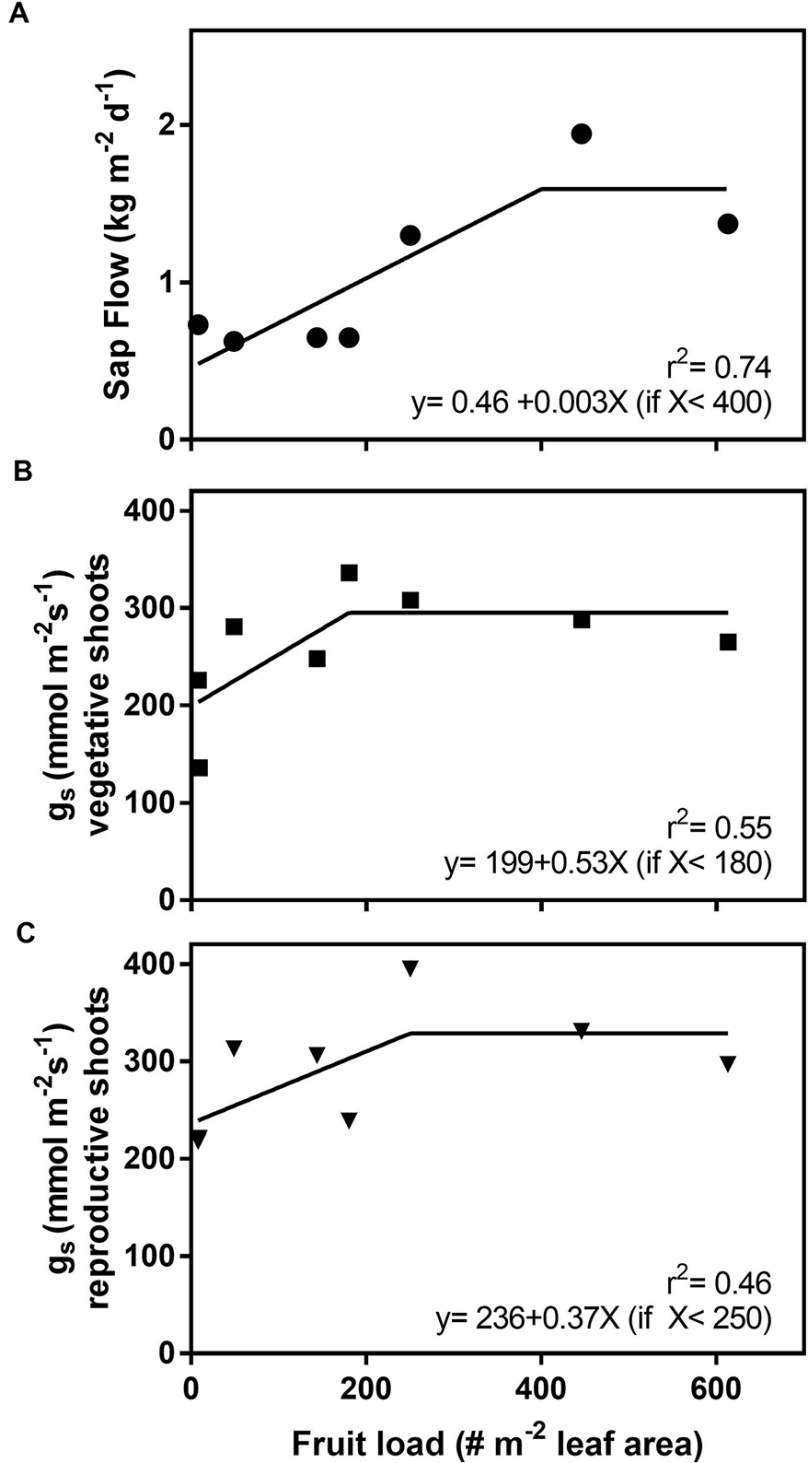
Daily sap flow **(A)**, stomatal conductance of leaves on vegetative shoots **(***g*_s_; **B)**, and stomatal conductance of leaves on reproductive shoots **(C)** as a function of fruit load in the independent fruit load experiment. Each point represents the average sap flow for 13 days for an individual tree with mean daily temperature ranging from 17°C to 27°C. Stomatal conductance was measured on March 1. A data point is not shown for the tree with 9.1 fruit m^−2^ leaf area in panel A due to a technical malfunction of the sap flow sensor. Data points shown for all trees are present in the other two panels.

Daily sap flow increased with fruit load up to a threshold of 400 fruit m^−2^ leaf area with no further increase above that value (**Figure 6A**). A rate of increase of 0.3 kg m^−2^ d^−1^ for each increase of 100 fruit m^−2^ leaf area occurred with sap flow being three times greater in trees at 400 or more fruit m^−2^ leaf area than those at minimal fruit loads. Similarly, *g*_s_ increased with fruit load up to 180 fruit m^−2^ in leaves on vegetative shoots and 250 fruit m^−2^ in leaves on reproductive shoots, with a higher dispersion of the data for reproductive shoots (**Figures 6B, C**). Differences in the fruit load values at which the plateau was reached for sap flow and *g*_s_ are likely attributable to the limited number of trees measured.

### Modeling Sap Flow Responses to Warming With Fruit Load

**Figure 7:**
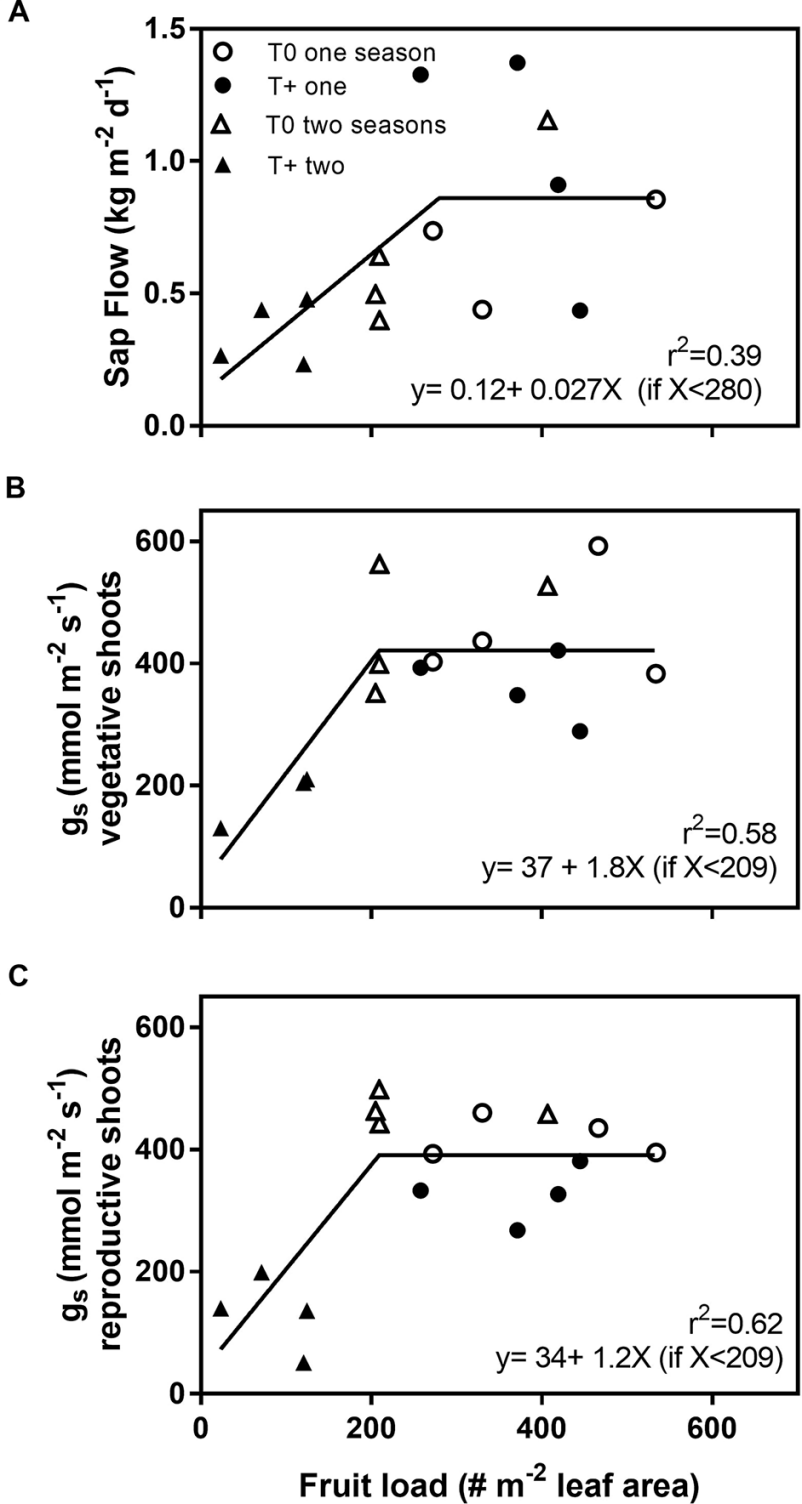
Daily sap flow **(A)**, stomatal conductance of leaves on vegetative shoots **(***g*_s_; **B)**, and stomatal conductance of leaves on reproductive shoots **(C)** as a function of fruit load in control (T0) and heated (T+) open-top chambers (OTCs). The trees were treated in the OTCs either one (2015–2016) or two seasons (2014–2015, 2015–2016). All trees were 3 years old during 2015–2016 when measurements were performed. Sap flow data correspond to the average of 9 (one season; March 14–22) and 24 (two seasons; March 23– April 17) measurement dates. Stomatal conductance is the average of two measurement dates (March 17, April 4) with similar weather conditions. Each point represents one tree per OTC.

Sap flow responses to warming during the later stages of fruit development (April) were reanalyzed using bilinear models to account for fruit load (**Figure 7**). Daily sap flow increased significantly with fruit load up to a threshold of 280 fruit m^−2^ leaf area (**Figure 7A**). Above this threshold, sap flow values were highly variable, which resulted in a modeled *r*^2^ value of 0.39 between fruit load and sap flow. In part, this was likely a consequence of sap flow being measured on different dates in one- and two-season trees (**Table 1**). Stomatal conductance, which was measured on the same dates in all trees, showed a similar bilinear function, but a much larger percentage of the variability was explained by fruit load (**Figures 7B, C**). The values of *r*^2^ were approximately 0.60 between fruit load and *g*_s_ for both vegetative and reproductive shoots.

## Discussion

This study employed an experimental approach for determining the water use of young olive trees under prolonged, elevated temperature (+4°C) conditions using OTCs. A modest increase in VPD accompanied the temperature increase in the heated OTCs as has been seen in other studies (Norby et al., 1997; Sadras et al., 2012a), but other microclimate variables such as PPFD were similar to the control OTCs. Our experimental approach is consistent with regional climate models, which predict that aridity will increase by midcentury in our Andean region (Penalba and Rivera, 2013; Zaninelli et al., 2019). The active heating system also allowed for maintaining the high level of heating necessary to obtain a +4°C temperature increase. More open systems using infrared heaters provide more natural microclimate conditions, but obtaining large temperature differences is difficult under windy conditions (Kimball et al., 2018).

Whole-tree sap flow in young olive trees was slightly higher in the heated than the control trees early in fruit development in January for both trees in the OTCs either one or two seasons (**Figures 3A, C**). Leaf photosynthetic gas exchange measurements also indicated higher transpiration rates in the T+ trees (Miserere, unpublished results). In grapevines, even a modest warming between 1°C and 2°C resulted in higher sap flow due to both greater leaf area and slightly higher VPD in heated vines than control vines under field conditions (Bonada et al., 2018). Aspen trees also showed significant increases in water use when heated by 5°C under growth chamber conditions because of greater growth and whole-tree hydraulic conductance in heated seedlings (Way et al., 2013). In the current study, leaf area per tree was similar between T+ and T0 trees (**Figure 2C**) most likely because the trees were heated either one or two seasons in the summer and fall when shoot growth was fairly low. Even in young olive trees, vegetative growth at that time of the year is very limited due to fruit growth and oil accumulation (Rosati et al., 2018). Also, root biomass did not show any differences between T0 and T+ trees (data not shown). Thus, it did not appear that differences in root biomass contributed to the sap flow responses.

The higher sap flow of the heated trees was explained by a single linear relationship with mean daily temperature early in fruit development (January) in both trees heated either one or two seasons (**Figures 4A, C**). Given that neither leaf area nor *g*_s_ (**Table 2**) was affected by heating for one season, it appears likely that elevated temperature accompanied by greater VPD led to the higher daily sap flow in these trees. In the trees heated for two seasons, *g*_s_ tended to be lower in January, although sap flow was somewhat higher in these same trees. Stomatal conductance has been shown to decrease with increasing instantaneous VPD values up to approximately 3.5 kPa in some previous olive field studies, but to be largely unresponsive to higher VPD values (Fernández et al., 1997; Rousseaux et al., 2008). In the present study, midday VPD values when *g*_s_ was measured were substantially higher than the 3.5 kPa threshold in both T+ and T0 trees. Thus, it is suggested that the decrease in *g*_s_ at midday may have been an early response to the low fruit load rather than VPD in the T+ trees. As will be discussed further, *g*_s_ often decreases with fruit load (Martín-Vertedor et al., 2011; Naor et al., 2013).

Later in fruit development (April), T+ trees had much higher diurnal values of sap flow than T0 trees for trees heated during the current season (i.e., one-season trees) (**Figure 3B**). Higher sap flow values in the T+ trees were even apparent for a given mean daily temperature (**Figure 4B**). This response could be explained by acclimation to temperature after several months of warming. In addition to greater tree sap flow, current season shoots of T+ trees had a higher total vessel number than the T0 trees due to more small-diameter vessels (**Figure 5A**). In olive, modifications in vessel density and diameter of current season shoots have been previously reported in response to deficit irrigation (Torres-Ruiz et al., 2013; Rossi et al., 2013), but not as a function of growth temperature. An increase in vessel number can be indicative of greater shoot water transport capacity in trees (Way et al., 2013), although this is not always the case. Further analysis shows that the theoretical specific hydraulic conductivity of the T+ trees was actually somewhat lower than that of the T0 trees based on the Hagen-Poiseuille equation (data not shown) (Tyree and Ewers, 1991). This occurred because the increase in vessel number in the T+ trees was due to more small-diameter vessels, which have little conductivity, and some tendency to have fewer large vessels that have very high conductivities. Thus, it does not appear that changes in vessel number in our study and their distribution resulted in the greater sap flow in the T+ trees. López-Bernal et al. (2010) also did not find a good correlation between the theoretical specific conductivity and sap velocity in mature olive trees. Other mechanisms in T+ trees such as lower leaf water potential due to osmotic adjustment as occurs under water stress in olive trees (Dichio et al., 2005; Lo Bianco and Scalisi, 2017) or less sap viscosity at higher temperature (Cochard et al., 2000) might explain the greater sap flow. Further research is needed in this regard. Lastly, the increase in small-diameter vessel number could suggest a safer water transport system based on redundancy and potentially less vulnerability to cavitation as reviewed by Hacke et al. (2017).

In contrast to the T+ trees heated for one season, sap flow was lower in T+ trees heated for two seasons compared to T0 trees late in fruit development for any given temperature (**Figure 4D**). The T+ trees had high fruit loads similar to the T0 trees the first season, but fruit load was much lower in the T+ trees than the controls the second season due to less flowering (**Figure 2B**). Moreover, total fruit dry weight was also lower in T+ than T0 (148 vs. 547 g tree^−1^) because of the combined effect of a lower fruit load and smaller individual fruit size (0.8 vs. 1.01 g fruit^−1^) due to the temperature treatment. Whole-tree water use using lysimeters has previously been shown to be low under low fruit loads with water use increasing markedly as fruit load increased, and the response to fruit load was much greater late in the season when fruit size was near maximum (Bustan et al., 2016). In the present study, the *g*_s_ was also lower in the T+ trees in April during the second season of heating. As mentioned previously, similar changes in *g*_s_ with fruit load have been reported in some other olive field studies (Martín-Vertedor et al., 2011; Naor et al., 2013), although this is not always the case (Proietti et al., 2006; Bustan et al., 2016). Sap flow and *g*_s_ in our study also showed a strong response to increasing fruit load when trees with a wide range of fruit loads were evaluated in an independent experiment in our field nursery (**Figure 6**).

A reanalysis of the warming experiment corroborated that fruit load explained a significant portion of the sap flow and *g*_s_ results in the warming experiment (**Figure 7**). Bilinear models best fit the relationship between sap flow and *g*_s_ with fruit load, although sap flow was variable at high fruit loads. Such information may be of use in predicting complex responses to global warming using simulation models in olive (Morales et al., 2016). One future scenario is that if flowering and subsequent fruit load are reduced by warming in olive trees (Vuletin Selak et al., 2014; Benlloch-González et al., 2018), warming would lead to less whole-tree transpiration and lower irrigation needs in olive orchards. Alternatively, different cultivars may respond differently to warming (De Ollas et al., 2019), and responses may vary due to regional climate. Such possibilities suggest a wider range of future scenarios.

## Conclusions

Sap flow of young olive trees appeared to increase due to elevated temperature and accompanying increases in VPD in the short term with further increases apparent over several months in the first season of warming. In contrast, trees heated for more than one season had lower fruit loads, which decreased sap flow in heated trees late in the season when fruit were near full size. These results provide a further understanding of the ecophysiological responses of olive trees to temperature and emphasize that multiple, interacting factors should be considered when predicting warming effects on water use in olive orchards.

## Data Availability Statement

The datasets generated for this study are available on request to the corresponding author.

## Author Contributions

AM, PS, and MR designed the measurements and sampling protocols. PM and GM performed the anatomical measurements. MR and AM performed most of the data collection, data processing, and statistical analyses. All authors contributed to the overall intellectual development of the study and the writing of the manuscript.

## Funding

This research was supported by grants from the Ministerio de Ciencia, Tecnología e Innovación Productiva de Argentina (ANPCyT, PICT2015 0195) and CONICET (PUE 2016 0125).

## Conflict of Interest

The authors declare that the research was conducted in the absence of any commercial or financial relationships that could be construed as a potential conflict of interest.

## References

[B1] Adams W. W. Stewart J. J. Cohu C. M. Muller O. Demmig-Adams B. (2016). Habitat temperature and precipitation of *Arabidopsis thaliana* ecotypes determine the response of foliar vasculature, photosynthesis, and transpiration to growth temperature. Front. Plant Sci. 7, 1026. 10.3389/fpls.2016.01026 27504111 PMC4959142

[B2] Allen R. G. Pereira L. S. Raes D. Smith M. (1998). Crop evapotranspiration: guidelines for computing crop water requirements. FAO Irrigation and Drainage Paper No. 56. Rome: FAO.

[B3] Aybar V. E. De Melo-Abreu J. P. Searles P. S. Matias A. C. Del Río C. Caballero J. M. (2015). Evaluation of olive flowering at low latitude sites in Argentina using a chilling requirement model. Spanish J. Agric. Res. 13, 1–10. 10.5424/sjar/2015131-6375

[B4] Ayerza R. Sibbett G. S. (2001). Thermal adaptability of olive (*Olea europaea* L.) to the Arid Chaco of Argentina. Agric. Ecosyst. Environ. 84, 277–285. 10.1016/S0167-8809(00)00260-7

[B5] Benlloch-González M. Sánchez-Lucas R. Aymen M. Benlloch M. Fernández-Escobar R. (2019). Global warming effects on yield and fruit maturation of olive trees growing under field conditions. Sci. Hortic. 249, 162–167. 10.1016/j.scienta.2019.01.046

[B6] Benlloch-González M. Sánchez-Lucas R. Benlloch M. Fernández-Escobar R. (2018). An approach to global warming effects on flowering and fruit set of olive trees growing under field conditions. Sci. Hortic. 240, 405–410. 10.1016/j.scienta.2018.06.054

[B7] Bonada M. Buesa I. Moran M. A. Sadras V. O. (2018). Interactive effects of warming and water deficit on Shiraz vine transpiration. OENO One 52, 189–202. 10.20870/oeno-one.2018.52.2.2141

[B8] Bustan A. Dag A. Yermiyahu U. Erel R. Presnov E. Agam N. (2016). Fruit load governs transpiration of olive trees. Tree Physiol. 36, 380–391. 10.1093/treephys/tpv138 26802540 PMC4885946

[B9] Chebbi W. Boulet G. Le Dantec V. Lili Chabaane Z. Fanise P. Mougenot B. (2018). Analysis of evapotranspiration components of a rainfed olive orchard during three contrasting years in a semi-arid climate. Agric. For. Meteorol. 257, 159–178. 10.1016/j.agrformet.2018.02.020

[B10] Cochard H. Martin R. Gross P. Bogeat-Triboulot M. B. (2000). Temperature effects on hydraulic conductance and water relations of *Quercus robur* L. J. Exp. Bot. 51, 1255–1259. 10.1093/jexbot/51.348.1255 10937701

[B11] Cuevas M. V. Martín-Palomo M. J. Díaz-Espejo A. Torres-Ruiz J. M. Rodriguez-Dominguez C. M. Perez-Martin A. (2013). Assessing water stress in a hedgerow olive orchard from sap flow and trunk diameter measurements. Irrig. Sci. 31, 729–746. 10.1007/s00271-012-0357-x

[B12] De Ollas C. Morillón R. Fotopoulos V. Puértolas J. Ollitrault P. Gómez-Cadenas A. (2019). Facing climate change: biotechnology of iconic Mediterranean woody crops. Front. Plant Sci. 10, 427. 10.3389/fpls.2019.00427 31057569 PMC6477659

[B13] Dichio B. Xiloyannis C. Sofo A. Montanaro G. (2005). Osmotic regulation in leaves and roots of olive trees during a water deficit and rewatering. Tree Physiol. 26, 179–185. 10.1093/treephys/26.2.179 16356914

[B14] Di Rienzo J. A. Casanoves F. Balzarini M. G. Gonzalez L. Tablada M. Robledo C. W. (2016). InfoStat versión 2016. Argentina: Grupo InfoStat, FCA, Universidad Nacional de Córdoba.

[B15] Fereres E. Orgaz F. Gonzalez-Dugo V. (2011). Reflections on food security under water scarcity. J. Exp. Bot. 62, 4079–4086. 10.1093/jxb/err165 21624976

[B16] Fernández J. E. Moreno F. Girón I. F. Blázquez O. M. (1997). Stomatal control of water use in olive tree leaves. Plant Soil 190, 179–192. 10.1023/A:1004293026973

[B17] Fernández J. E. Durán P. J. Palomo M. J. Diaz-Espejo A. Chamorro V. Girón I. F. (2006). Calibration of sap flow estimated by the compensation heat pulse method in olive, plum and orange trees: relationships with xylem anatomy. Tree Physiol. 26, 719–728. 10.1093/treephys/26.6.719 16510387

[B18] Fernández J. E. Torrecillas A. (2012). For a better use and distribution of water: an introduction. Agric. Water Manage. 114, 1–3. 10.1016/j.agwat.2012.07.004

[B19] García-Inza G. P. Castro D. N. Hall A. J. Rousseaux M. C. (2014). Responses to temperature of fruit dry weight, oil concentration, and oil fatty acid composition in olive (*Olea europaea* L. var. ‘Arauco’). Eur. J. Agron. 54, 107–115. 10.1016/j.eja.2013.12.005

[B20] García-Inza G. P. Castro D. N. Hall A. J. Rousseaux M. C. (2016). Opposite oleic acid responses to temperature in oils from the seed and mesocarp of the olive fruit. Eur. J. Agron. 76, 138–147. 10.1016/j.eja.2016.03.003

[B21] Gómez-del-Campo M. Morales-Sillero A. Vita Serman F. Rousseaux M. C. Searles P. S. (2010). Olive growing in the arid valleys of northwest Argentina (provinces of Catamarca, La Rioja and San Juan). Olivae 114, 23–45.

[B22] Grossman Y. L. DeJong T. M. (1994). PEACH: a simulation model of reproductive and vegetative growth in peach trees. Tree Physiol. 14, 329–345. 10.1093/treephys/14.4.329 14967690

[B23] Hacke U. G. Spicer R. Schreiber S. G. Plavcová L. (2017). An ecophysiological and developmental perspective on variation in vessel diameter. Plant Cell Environ. 40, 831–845. 10.1111/pce.12777 27304704

[B24] Haworth M. Marino G. Brunetti C. Killi D. De Carlo A. Centritto M. (2018). The impact of heat stress and water deficit on the photosynthetic and stomatal physiology of olive (*Olea europaea* L.)—a case study of the 2017 heat wave. Plants 7, 1–13. 10.3390/plants7040076 PMC6313851 30241389

[B25] Hernández-Santana V. Fernández J. E. Rodriguez-Dominguez C. M. Romero R. Díaz-Espejo A. (2016). The dynamics of radial sap flux density reflects changes in stomatal conductance in response to soil and air water deficit. Agric. For. Meteorol. 218 (219), 92–101. 10.1016/j.agrformet.2015.11.013

[B26] IPCC (2014). Climate change 2014: synthesis report. Contribution of Working Groups I, II and III to the fifth assessment report of the Intergovernmental Panel on Climate Change. Geneva, Switzerland: IPCC.

[B27] Jones H. (1998). Stomatal control of photosynthesis and transpiration. J. Exp. Bot. 49, 387–398. 10.1093/jxb/49.Special_Issue.387

[B28] Kellomäki S. Wang K. Y. (2000). Modelling and measuring transpiration from Scots pine with increased temperature and carbon dioxide enrichment. Ann. Bot. 85, 263–278. 10.1006/anbo.1999.1030 PMC4240391 12234144

[B29] Kimball B. A. Alonso-Rodríguez A. M. Cavaleri M. A. Reed S. C. González G. Wood T. E. (2018). Infrared heater system for warming tropical forest understory plants and soils. Ecol. Evol. 8, 1932–1944. 10.1002/ece3.3780 29468013 PMC5817131

[B30] Lo Bianco R. Scalisi A. (2017). Water relations and carbohydrate partitioning of four greenhouse-grown olive genotypes under long-term drought. Trees 31, 717–727. 10.1007/s00468-016-1502-6

[B31] López-Bernal A. Alcántara E. Testi L. Villalobos F. J. (2010). Spatial sap flow and xylem anatomical characteristics in olive trees under different irrigation regimes. Tree Physiol. 30, 1536–1544. 10.1093/treephys/tpq095 21081652

[B32] López-Bernal A. García-Tejera O. Testi L. Orgaz F. Villalobos F. J. (2018). Stomatal oscillations in olive trees: analysis and methodological implications. Tree Physiol. 38, 531–542. 10.1093/treephys/tpx127 29040757

[B33] Lorite I. J. Gabaldón-Leal C. Ruiz-Ramos M. Belaj A. de la Rosa R. León L. (2018). Evaluation of olive response and adaptation strategies to climate change under semi-arid conditions. Agric. Water Manage. 204, 247–261. 10.1016/j.agwat.2018.04.008

[B34] Mailer R. J. Ayton J. Graham K. (2010). The influence of growing region, cultivar and harvest timing on the diversity of Australian olive oil. J. Am. Oil Chem. Soc. 87, 877–884. 10.1007/s11746-010-1608-8

[B35] Martín-Vertedor A. I. Pérez-Rodríguez J. M. Prieto Losada H. Fereres Castiel E. (2011). Interactive responses to water deficits and crop load in olive (*Olea europaea* L., cv. Morisca) I.—growth and water relations. Agric. Water Manage. 98, 941–949. 10.1016/j.agwat.2011.01.002

[B36] Maseda P. H. Fernández R. J. (2006). Stay wet or else: three ways in which plants can adjust hydraulically to their environment. J. Exp. Bot. 57, 3963–3977. 10.1093/jxb/erl127 17079697

[B37] McCulloh K. A. Petitmermet J. Stefanski A. Rice K. E. Rich R. L. Montgomery R. A. (2016). Is it getting hot in here? Adjustment of hydraulic parameters in six boreal and temperate tree species after 5 years of warming. Glob. Chang. Biol. 22, 4124–4133. 10.1111/gcb.13323 27122300

[B38] Miserere A. Searles P. S. García-Inza G. P. Rousseaux M. C. (2018). Elevated temperature affects vegetative growth and fruit oil concentration in olive trees (*Olea europaea*). Acta Hortic. 1199, 523–528. 10.17660/ActaHortic.2018.1199.83

[B39] Miserere A. Searles P. S. Hall A. J. García-Inza G. P. Rousseaux M. C. Complementary active heating methods for evaluating the responses of young olive trees to warming. Sci. Hortic (in press) (2019). 257, 108754. 10.1016/j.scienta.2019.108754

[B40] Morales A. Leffelaar P. A. Testi L. Orgaz F. Villalobos F. J. (2016). A dynamic model of potential growth of olive (*Olea europaea* L.) orchards. Eur. J. Agron. 74, 93–102. 10.1016/j.eja.2015.12.006

[B41] Moriana A. Villalobos F. J. Fereres E. (2002). Stomatal and photosynthetic responses of olive (*Olea europaea* L.) leaves to water deficits. Plant Cell Environ. 25, 395–405. 10.1046/j.0016-8025.2001.00822.x

[B42] Naor A. Naschitz S. Peres M. Gal Y. (2008). Responses of apple fruit size to tree water status and crop load. Tree Physiol. 28, 1255–1261. 10.1093/treephys/28.8.1255 18519256

[B43] Naor A. Schneider D. Ben-Gal A. Zipori I. Dag A. Kerem Z. (2013). The effects of crop load and irrigation rate in the oil accumulation stage on oil yield and water relations of ‘Koroneiki’ olives. Irrig. Sci. 31, 781–791. 10.1007/s00271-012-0363-z

[B44] Norby R. J. Edwards N. T. Riggs J. S. Abner C. H. Wullschleger S. D. Gunderson C. A. (1997). Temperature-controlled open-top chambers for global change research. Glob. Chang. Biol. 3, 259–267. 10.1046/j.1365-2486.1997.00072.x

[B45] Orgaz F. Villalobos F. J. Testi L. Fereres E. (2007). A model of daily mean canopy conductance for calculating transpiration of olive canopies. Funct. Plant Biol. 34, 178–188. 10.1071/FP06306 32689344

[B46] Penalba O. Rivera J. A. (2013). Future changes in drought characteristics over southern South America projected by a CMIP5 multi-model ensemble. Am. J. Clim. Change 2, 173–182. 10.4236/ajcc.2013.23017

[B47] Proietti P. Nasini L. Famiani F. (2006). Effect of different leaf-to-fruit ratios on photosynthesis and fruit growth in olive (*Olea europaea* L.). Photosynthetica 44, 275–285. 10.1007/s11099-006-0019-4

[B48] Rondanini D. P. Castro D. N. Searles P. S. Rousseaux M. C. (2014). Contrasting patterns of fatty acid composition and oil accumulation during fruit growth in several olive varieties and locations in a non-Mediterranean region. Eur. J. Agron. 52, 237–246. 10.1016/j.eja.2013.09.002

[B49] Rosati A. Paoletti A. Al Hariri R. Morelli A. Famiani F. (2018). Resource investments in reproductive growth proportionately limit investments in whole-tree vegetative growth in young olive trees with varying crop loads. Tree Physiol. 38, 1–11. 10.1093/treephys/tpy011 29474732

[B50] Rossi L. Sebastiani L. Tognetti R. Andria R. Morelli G. Cherubini P. (2013). Tree-ring wood anatomy and stable isotopes show structural and functional adjustments in olive trees under different water availability. Plant Soil 372, 567–579. 10.1007/s11104-013-1759-0

[B51] Rousseaux M. C. Benedetti J. P. Searles P. S. (2008). Leaf-level responses of olive trees (*Olea europaea*) to the suspension of irrigation during the winter in an arid region of Argentina. Sci. Hortic. 115, 135–141. 10.1016/j.scienta.2007.08.005

[B52] Rousseaux M. C. Figuerola P. I. Correa-Tedesco G. Searles P. S. (2009). Seasonal variations in sap flow and soil evaporation in an olive (*Olea europaea* L.) grove under two irrigation regimes in an arid region of Argentina. Agric. Water Manage. 96, 1037–1044. 10.1016/j.agwat.2009.02.003

[B53] Sadras V. O. Bubner R. Moran M. A. (2012a). A large-scale, open-top system to increase temperature in realistic vineyard conditions. Agric. For. Meteorol. 154 (155), 187–194. 10.1016/j.agrformet.2011.11.005

[B54] Sadras V. O. Montoro A. Moran M. A. Aphalo P. J. (2012b). Elevated temperature altered the reaction norms of stomatal conductance in field-grown grapevine. Agric. For. Meteorol. 165, 35–42. 10.1016/j.agrformet.2012.06.005

[B55] Searles P. S. Agüero Alcarás M. Rousseaux M. C. (2011). Consumo del agua por el cultivo del olivo (*Olea europaea* L.) en el Noroeste de Argentina: una comparación con la Cuenca Mediterránea. Ecol. Aus. 21, 15–28.

[B56] Tanasijevic L. Todorovic M. Pereira L. S. Pizzigalli C. Lionello P. (2014). Impacts of climate change on olive crop evapotranspiration and irrigation requirements in the Mediterranean region. Agric. Water Manage. 144, 54–68. 10.1016/j.agwat.2014.05.019

[B57] Tognetti R. Giovannelli A. Lavini A. Morelli G. Fragnito F. D’Andria R. (2009). Assessing environmental controls over conductances through the soil-plant-atmosphere continuum in an experimental olive tree plantation of southern Italy. Agric. For. Meteorol. 149, 1229–1243. 10.1016/j.agrformet.2009.02.008

[B58] Torres M. Pierantozzi P. Searles P. Rousseaux M. C. García-Inza G. Miserere A. (2017). Olive cultivation in the southern hemisphere: flowering, water requirements and oil quality responses to new crop environments. Front. Plant Sci. 8, 1–12. 10.3389/fpls.2017.01830 29163569 PMC5663689

[B59] Torres-Ruiz J. M. Diaz-Espejo A. Morales-Sillero A. Martín-Palomo M. J. Mayr S. Beikircher B. (2013). Shoot hydraulic characteristics, plant water status and stomatal response in olive trees under different soil water conditions. Plant Soil 373, 77–87. 10.1007/s11104-013-1774-1

[B60] Tyree M. T. Ewers F. W. (1991). The hydraulic architecture of trees and other woody plants. New Phytol. 119, 345–360. 10.1111/j.1469-8137.1991.tb00035.x

[B61] Uceda M. Hermoso M. (2001). “La calidad del aceite de oliva,” in El cultivo del olivo. Eds. Barranco D. Fernández-Escobar R. Rallo L. (Madrid: Ediciones Mundi-Prensa), 589–614.

[B62] Urban J. Ingwers M. McGuire M. A. Teskey R. O. (2017). Stomatal conductance increases with rising temperature. Plant Signal. Behav. 12, 10.1080/15592324.2017.1356534 PMC5616154 28786730

[B63] Vuletin Selak G. Cuevas J. Goreta Ban S. Pinillos V. Dumicic G. Perica S. (2014). The effect of temperature on the duration of the effective pollination period in ‘Oblica’ olive (*Olea europaea*) cultivar. Ann. Appl. Biol. 164, 85–94. 10.1111/aab.12082

[B64] Way D. A. Domec J. Jackson R. B. (2013). Elevated growth temperatures alter hydraulic characteristics in trembling aspen (*Populus tremuloides*) seedlings: implications for tree drought tolerance. Plant Cell Environ. 36, 103–115. 10.1111/j.1365-3040.2012.02557.x 22690910

[B65] Zaninelli P. G. Menéndez C. G. Falco M. Franca N. L. Carril A. F. (2019). Future hydroclimatological changes in South America based on an ensemble of regional climate models. Clim. Dyn. 52, 819–830. 10.1007/s00382-018-4225-0

[B66] Zarlavsky G. E. (2014). Histología Vegetal: técnicas simples y complejas. Buenos Aires: Sociedad Argentina de Botánica.

